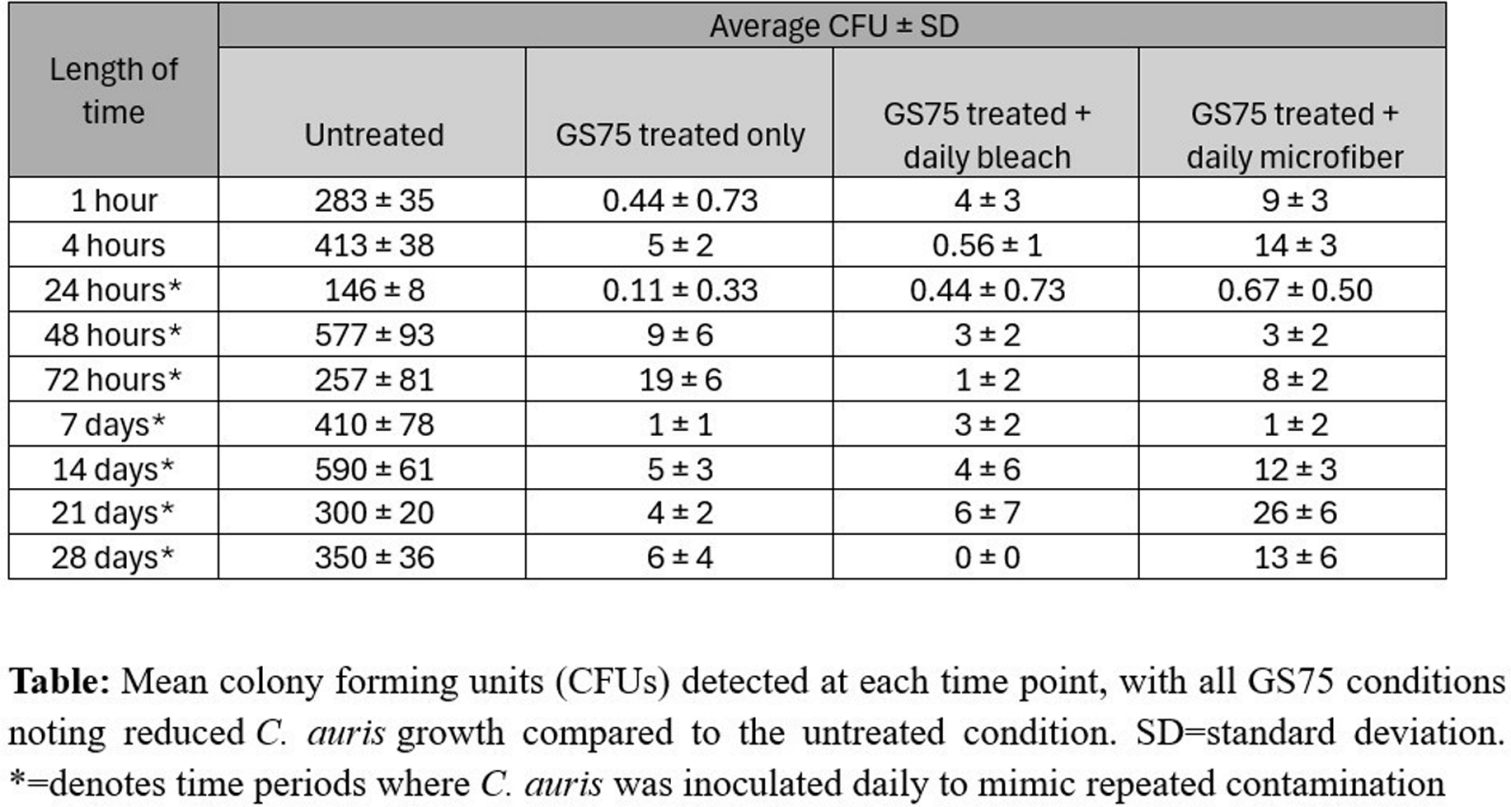# 225 Minocycline Activity Against Carbapenem Resistant Acinetobacter baumannii Isolates in a Large Integrated Healthcare System

**DOI:** 10.1017/ash.2026.10605

**Published:** 2026-06-23

**Authors:** Rachel Medernach, Mackenzie May, Ellen Gough, Sarah Sansom, Mary Hayden

**Affiliations:** 1 Rush University Medical Center; 2 Rush University

## Abstract

**Background:** Candidozyma auris is an emerging healthcare-associated pathogen that colonizes human skin and survives on healthcare surfaces for up to two weeks. As C. auris re-contaminates the healthcare environment within hours of disinfection, a continuously active disinfectant may provide an advantage over traditional disinfectants in healthcare environments with high rates of C. auris colonization. Water-stable organosilanes (WSOs) have demonstrated continuously active disinfection against bacterial and viral species but have not been studied against C. auris. Goldshield® GS75 (Locust Valley, NY) is a WSO composed of a siloxane bonding agent providing surface coating, a nitrogen molecule for organism attraction, a long carbon chain for cellular penetrance, and a quaternary ammonium compound for disinfection. We tested the efficacy and duration of effect of GS75 on survival of C. auris in vitro. Methods GS75 was applied to C. auris colony forming units (CFUs). After one hour, coupons were swabbed using premoistened sponge-sticks (Neogen Sponge-Stick with proprietary neutralizing buffer active against chlorine and QACs; Neogen, Lansing, MI), processed using the stomacher method and plated quantitatively to Sabouraud Dextrose agar (RemelTM, Lenexa, KS) then incubated for 48 hours at 37°C in ambient air. Duration of GS75 activity was assessed using the same method, with C. auris re-inoculated daily to simulate repeated contamination from a C. auris colonized or infected patient, and recovery attempted at 4 hours, 24 hours, 48 hours, 72 hours, 7 days, 14 days, 21 days, and 28 days after coupon treatment. (Figure). A separate set of coupons was wiped daily with a dry microfiber cloth or a 10% sodium hypochlorite bleach wipe (Sani-Cloth®, PDI, Woodcliff Lake, NJ) before daily re-inoculation to simulate routine environmental cleaning practices that might be necessary even with use of a continuously active disinfectant. Results Mean C. auris CFUs recovered from untreated coupons exceeded 145 at all sampling times, while GS75-treated coupons showed significantly reduced C. auris recovery. The addition of daily bleach or microfiber cloth wiping had minimal effect on GS75 activity (Table). Conclusion One application of GS75 reduced survival of C. auris on a common healthcare facility surface material for up to 28 days, despite repeated C. auris challenge and with minimal reduction in activity after physical or chemical cleaning. These findings support evaluation

**Figure:**
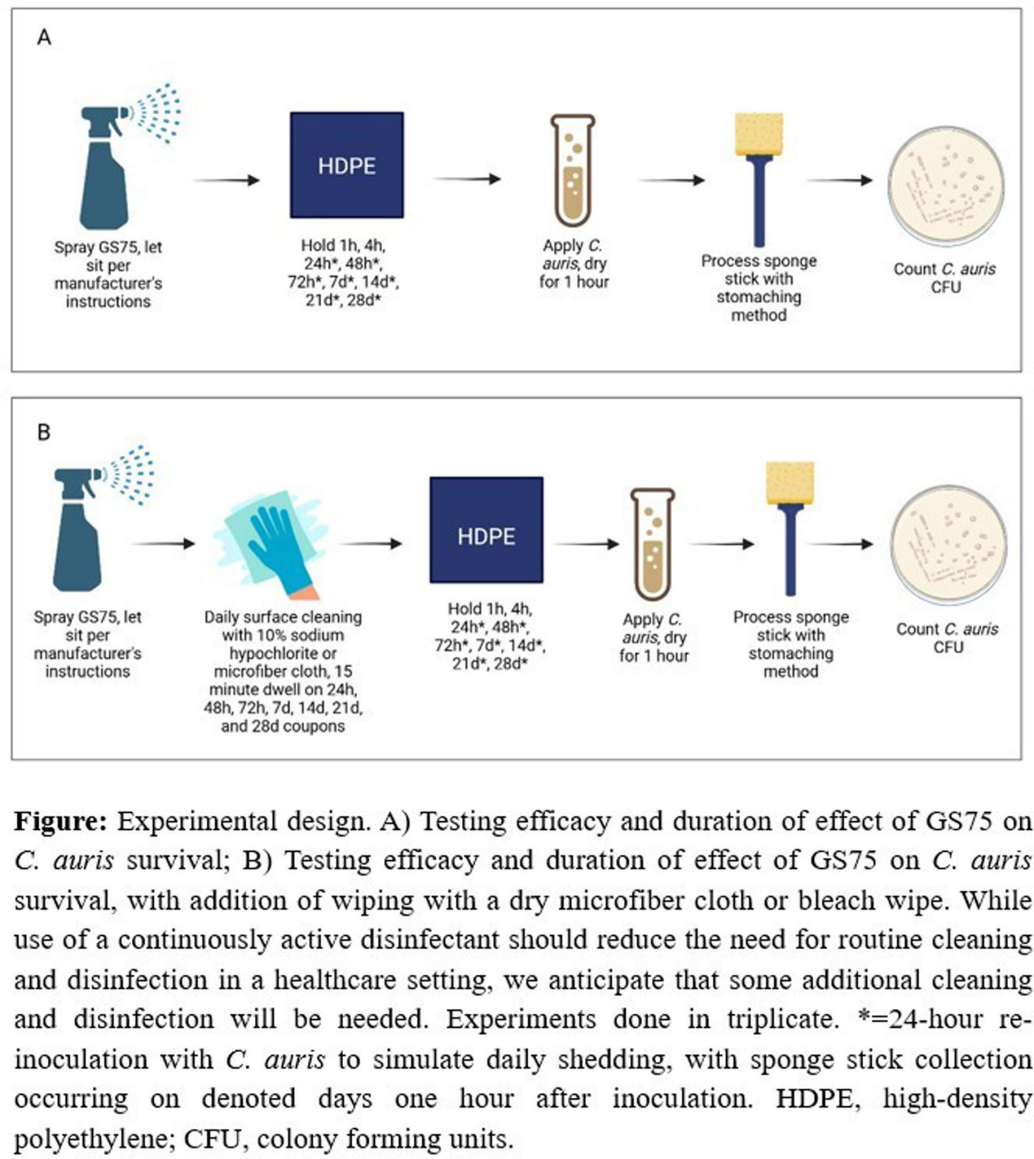

**Figure:**